# Supplementary material for: Wnt5A Signaling Blocks Progression of Experimental Visceral Leishmaniasis
Source: Front Immunol. 2022 Feb 7;13:818266. doi: 10.3389/fimmu.2022.818266 (PMC8859155; doi:10.3389/fimmu.2022.818266)
Supplement: Supplementary file 1 [file DataSheet_1.pdf]

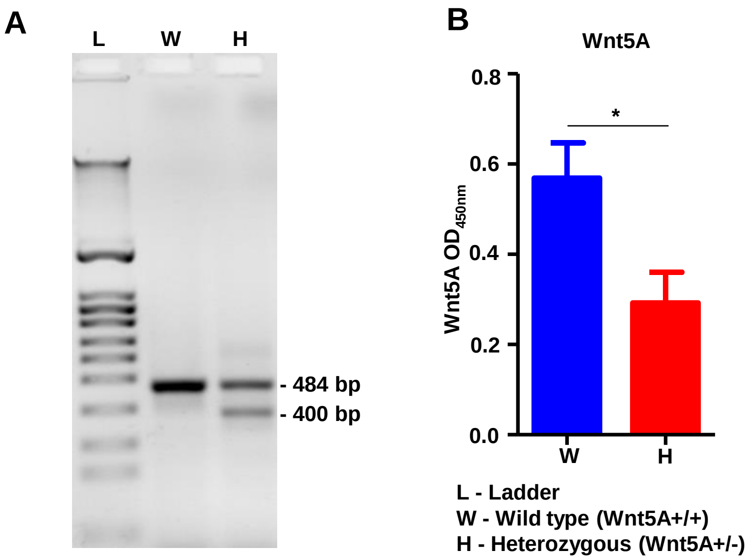

Figure S1: Characterization of Wnt5A heterozygous mice. Panel A: Identification of Wnt5A+/- (heterozygous) mouse by genotyping of DNA extracted from tail by PCR, following Jackson Laboratory protocol. Panel B: Graphical representation of relatively less Wnt5A protein in the plasma of Wnt5A heterozygous (H) mice as compared to wild type (W), as demonstrated by ELISA. Statistical analysis was performed with the unpaired t-test. Data are presented as mean ± SEM. Significance was annotated as follows: *$p \leq 0.05$, n=7 (per group).

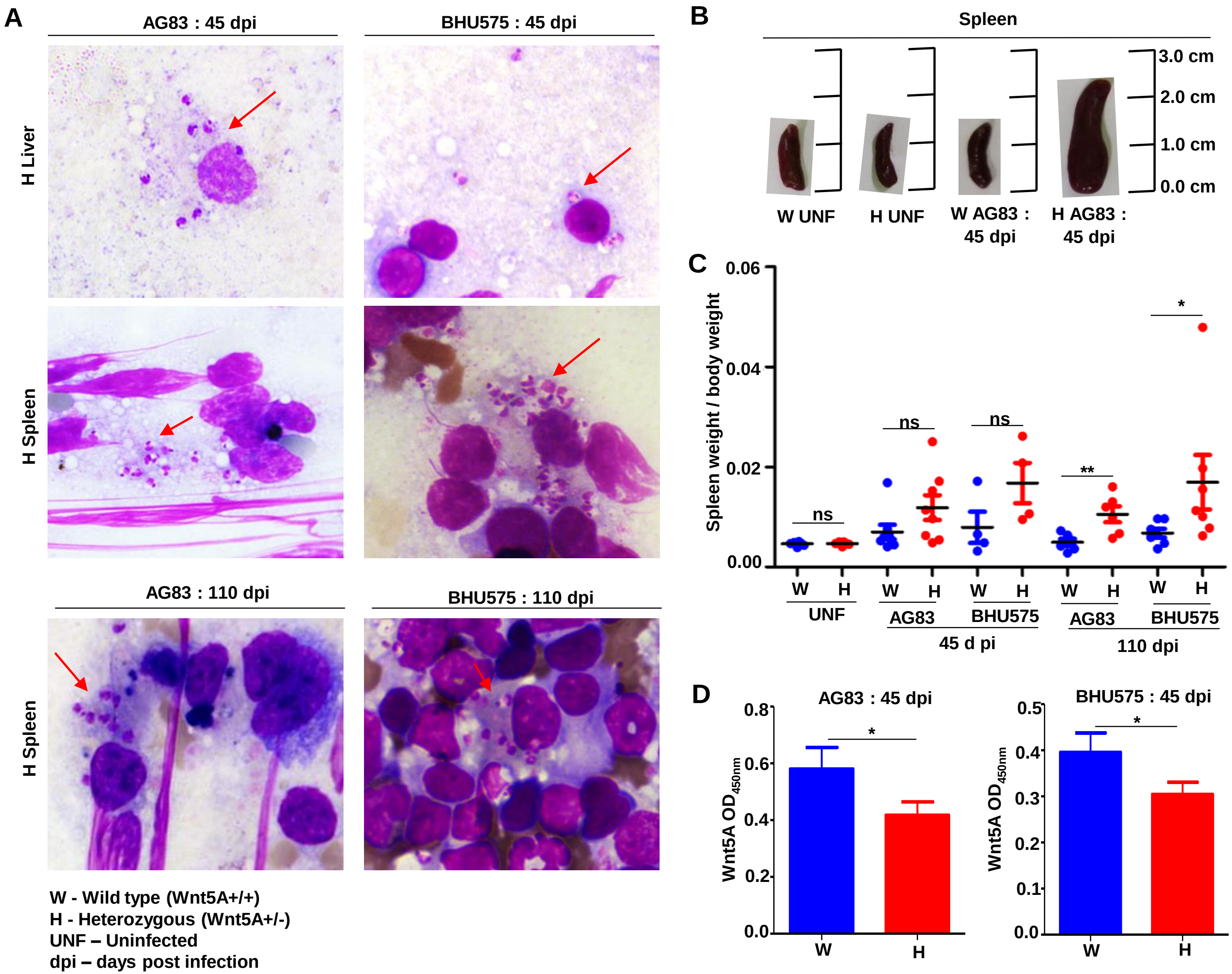

Figure S2: Wnt5A heterozygous (H) mice show significantly more *L. donovani* infection correlating with high frequency of splenomegaly as compared to wild type (W) controls. Panel A: Representative Giemsa stained micrographs of imprints (under 100X objective/oil immersion) demonstrating *L. donovani* infection (arrows) in Wnt5A+/- (H). Panel B: Representation of calibration of spleen size. Panel C: Graphical representation of spleen weight in the H category as compared to W, after infection. Panel D: H category of mice retain lower level of plasma Wnt5A than the W category 45 days post *L. donovani* infection. Statistical analysis was performed with the unpaired t-test. Data are presented as mean ± SEM. Significance was annotated as follows: $*p \leq 0.05$, ns: not significant, n=4 to 8 (per group).

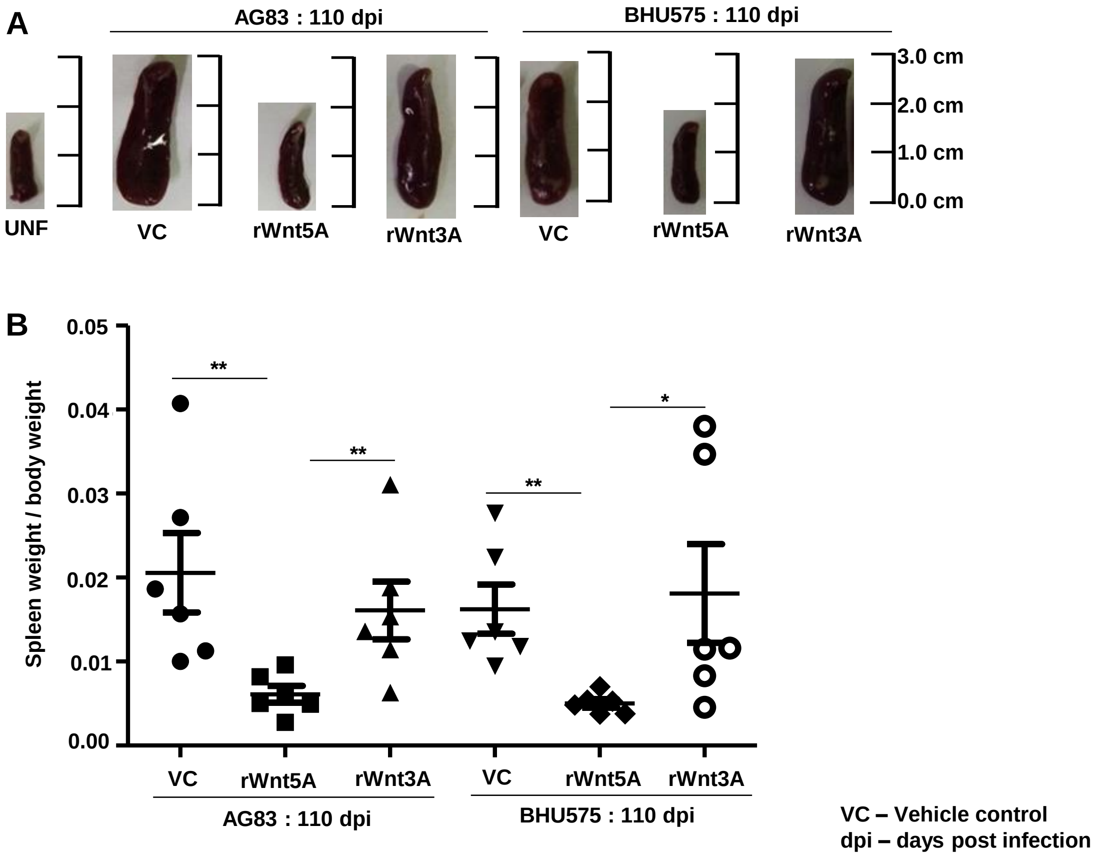

Figure S3: Incidence of *L. donovani* infection induced splenomegaly is lower in rWnt5A treated mice than in the corresponding controls. Panel A: Representation of calibration of spleen size in infected mice pretreated with rWnt5A/rWnt3A/VC. Panel B: Graphical representation of spleen weight in rWnt5A, rWnt3A or VC treated mice demonstrating reduced frequency of splenomegaly in rWnt5A treated infected mice as compared to those treated with rWnt3A or VC. Statistical analysis was performed with the unpaired t-test. Data are presented as mean ± SEM. Significance was annotated as follows: *$p \leq 0.05$, **$p \leq 0.005$, n=6 (per group).

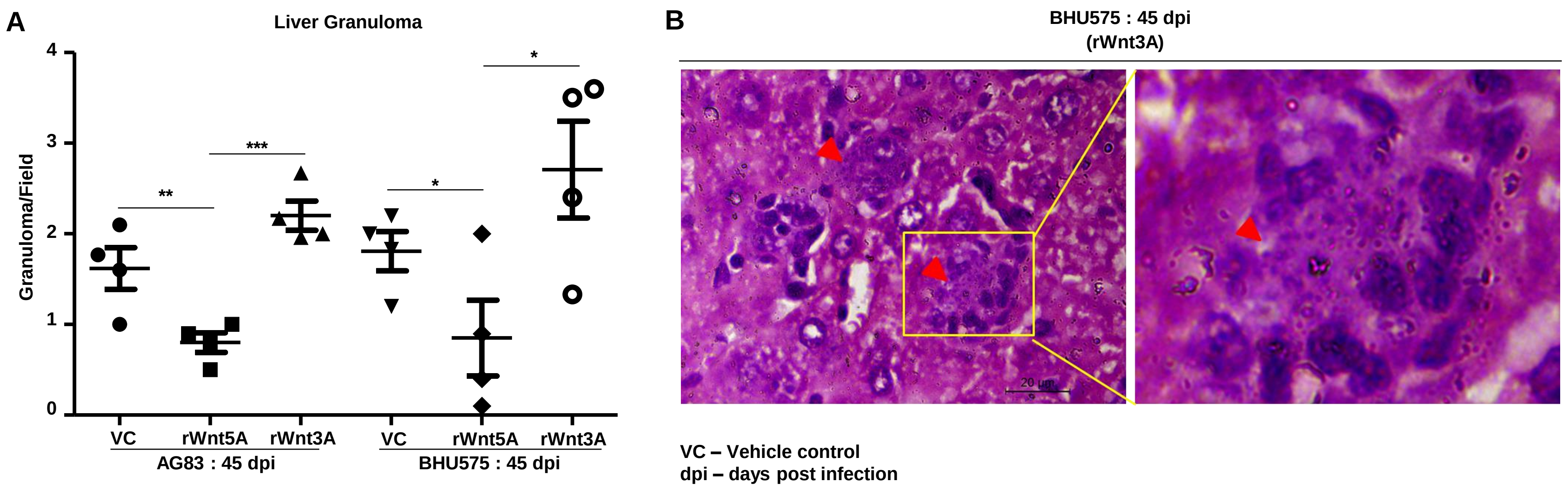

Figure S4: <u>Frequency of liver granuloma is significantly less in *L. donovani* infected mice pretreated with rWnt5A, but not rWnt3A or VC</u>. Panel A: Graphical representation of reduced frequency of liver granuloma subject to rWnt5A pretreatment of infected mice as opposed to pretreatment with rWnt3A or VC. B: Micrograph of H & E-stained liver section (under 100X objective/oil) demonstrating the presence of parasites inside the liver granuloma (inset) of an rWnt3A pretreated BHU575 infected mouse. Statistical analysis was performed with the unpaired t-test. Data are presented as mean ± SEM. Significance was annotated as follows: *$p \leq 0.05$, **$p \leq 0.005$, ***$p \leq 0.005$, n=4 (per group).

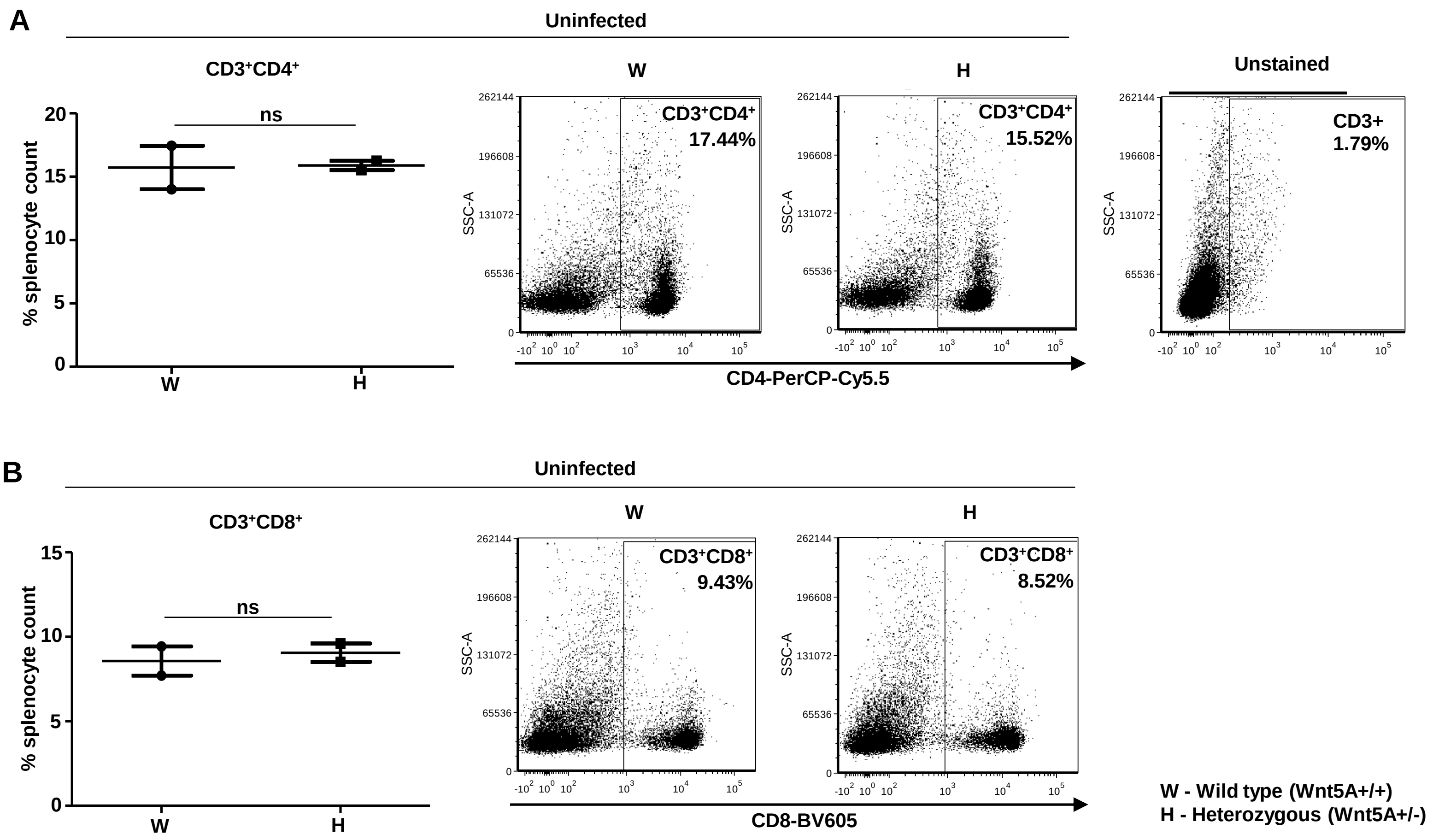

Figure S5: <u>Comparison of percentage of $CD3^+CD4^+$ and $CD3^+CD8^+$ lymphocytes in Wnt5A heterozygous (H) and wild type (W) mice :</u> Panel A and B: Difference between the percentage of $CD3^+CD4^+$ cells (A) and $CD3^+CD8^+$ cells (B) in the H and W category of mice is not significant. FACS dot plots are showing the level of CD4+ and CD8+ cells (gated on CD3+ cells using unstained as reference) in respective categories. Statistical analysis was performed as unpaired t-test. Statistical analysis was performed with the unpaired t-test. Data are presented as mean ± SEM. Significance was annotated as follows: ns: not significant, n=2 (per group).

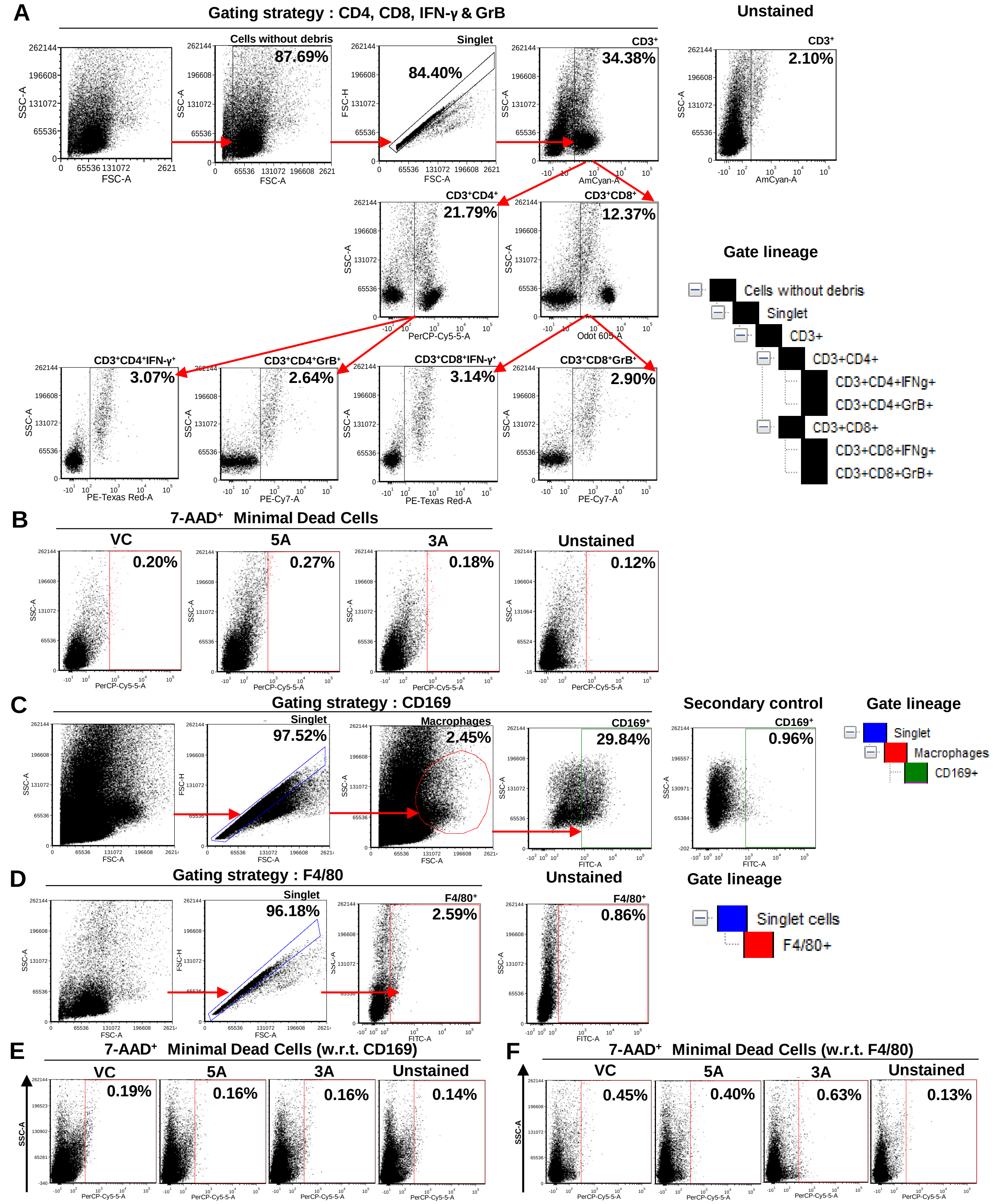

Figure S6: Representation of FACS gating strategy (Figure 6 and Figure 7) : Panel A: Gating strategy for analysis of CD3+CD4+ and CD3+CD8+ cells that are positive for IFN-γ and GrB. Panel B: FACS representation of 7-AAD stained splenocytes harvested separately from rWnt5A/rWnt3A/vc pretreated infected mice after brefeldin treatment, showing minimal cell death. Panel C and D: Analysis of gating strategy for CD169 (Panel C) and F4/80 (Panel D) positive cells. Panel E and F: FACS representation of 7-AAD stained splenocytes harvested separately from rWnt5A/rWnt3A/vc pretreated infected mice just before data acquisition showing minimal cell death.

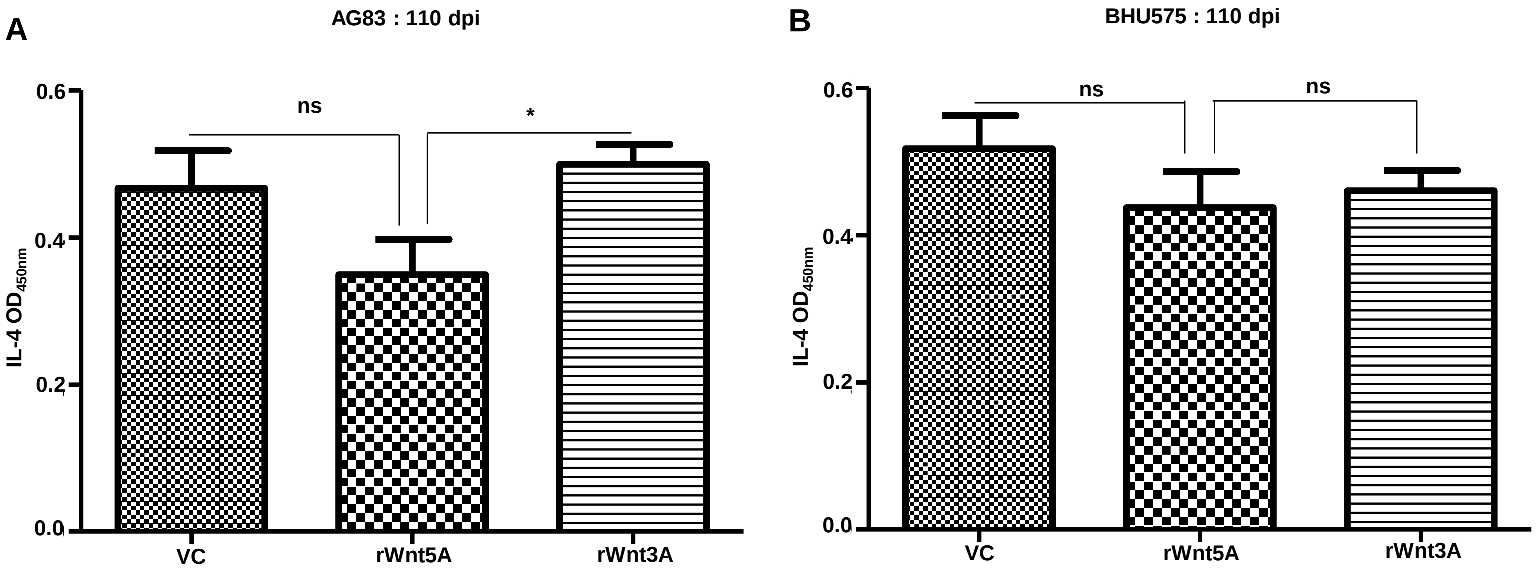

Figure S7: Analysis of plasma IL-4 in rWnt5A/rWnt3A/VC pretreated mice infected with *L. donovani* AG83 or BHU575. ELISA demonstrating the relative levels of IL-4 in the plasma of rWnt5A/rWnt3A/VC pretreated mice infected with either AG83 (Panel A) or BHU575 (Panel B). Statistical analysis was performed with the unpaired t-test. Data are presented as mean ± SEM. Significance was annotated as follows: *$p \leq 0.05$, ns: not significant, n=6 (per group).

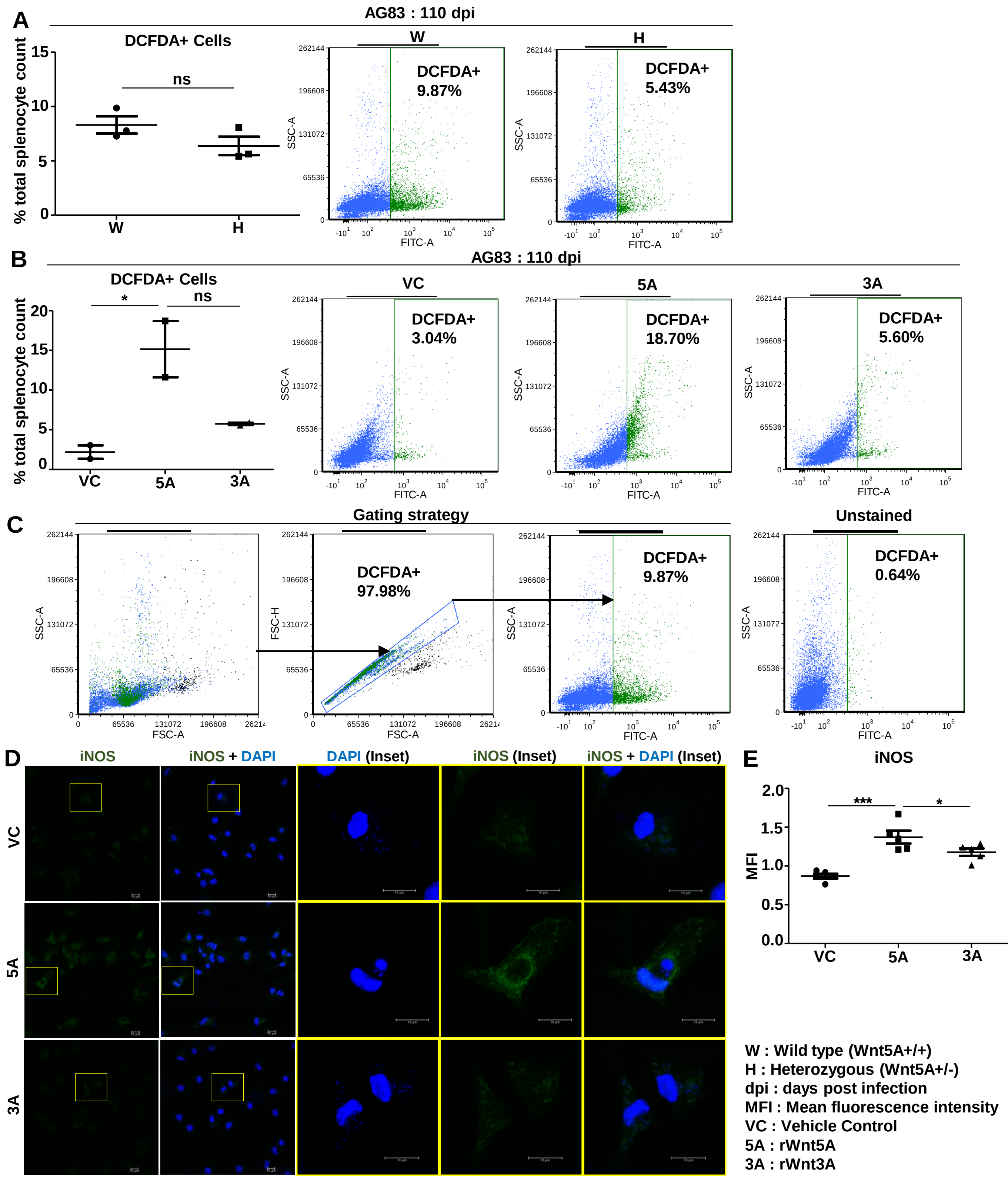

Figure S8: Wnt5A dependent ROS / iNOS induction : Panel A and B: Increased percentage of DCFDA$^+$ cells in the spleens of wild type (W) mice as compared to Wnt5A heterozygous (H) mice (Panel A) and rWnt5A pretreated mice as compared to controls (Panel B) after AG83 infection. Panel C: Gating strategy. Panel D: Confocal microscopy demonstrating increased iNOS expression in AG83 infected peritoneal macrophages as compared to controls. Panel E: Graphical representation of iNOS MFI as measured by ImageJ. Statistical analysis was performed with the unpaired t test. Data are presented as mean ± SEM. Significance was annotated as follows: $*p \leq 0.05$, $***p \leq 0.0005$, ns: not significant, n=2 to 3 (per group).

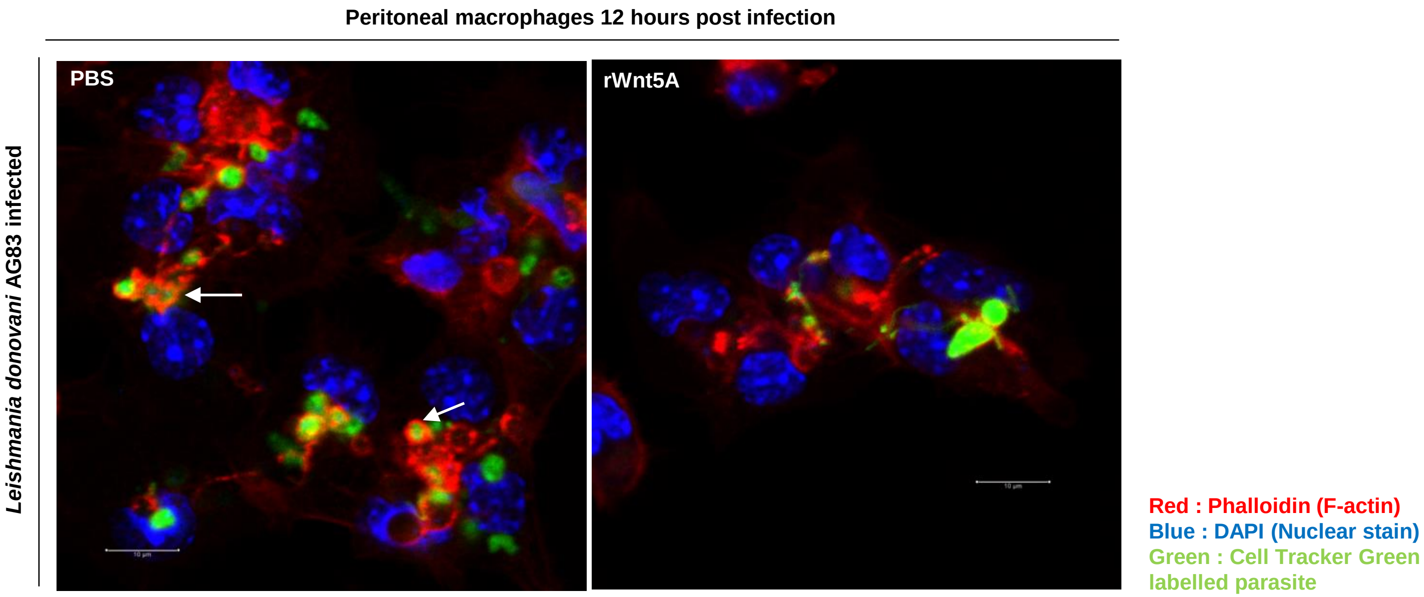

Figure S9: Wnt5A associated restriction of *L. donovani* infection correlates with remodeling of the actin cytoskeleton. Confocal microscopy of phalloidin stained peritoneal macrophages infected with *L. donovani* demonstrates rWnt5A treatment associated disruption of actin rings that are otherwise present after infection. Arrow marks denote actin rings (under 63X objective/oil immersion/2.51 zoom).

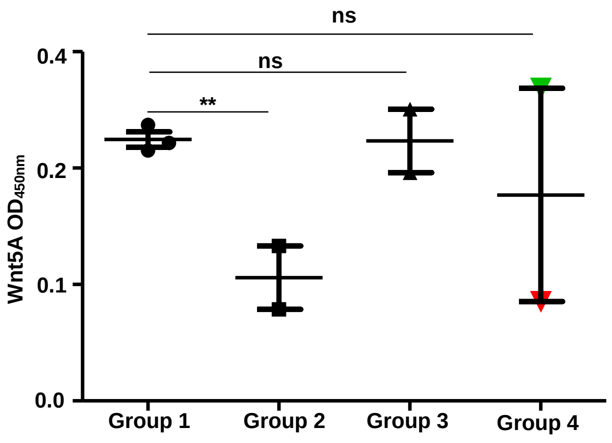

Group 1 – Healthy Individuals
Group 2 – rK39- with lesions
Group 3 – rK39+ with lesions and under Miltefosine treatment
Group 4 – rK39+ without lesion, no treatment

Figure S10: Varied level of Wnt5A in the plasma of healthy individuals and those with history of visceral leishmaniasis (VL) or post kala-azar visceral leishmaniasis (PKDL). Graphical representation of varied level of plasma Wnt5A in healthy individuals and those diagnosed with VL/PKDL based on reactivity to rK39 antibody. Patient history is explained in Table 1. Parasite clearance may be associated with the level plasma Wnt5A. Statistical analysis was performed with the unpaired t-test. Data are presented as mean ± SEM. Significance was annotated as follows: ** $p \leq 0.005$, ns: not significant, n=2 to 3 (per group).

Sample Number	Past symptoms	Current symptoms	Anti-Leishmanial antibody test (rK39 positve / negative)	Treatment taking
Sample 1 **(Group 3)**		Hypopigmented skin rash all over the body. No fever, no organomegaly	+	Miltefosine for about 2 months 20 days
Sample 2 **(Group 2)**	She was diagnosed on 27/07/2018. Skin scraping test was positive for LD bodies. On 27/07/2018, Hb was 7.8 g/dL (at disease onset), WBC count was 7,180.	Treated case with nearly complete recovery. Small lesion was found near her lower lip.	-	Treatment with Miltefosine completed on 2018
Sample 3 **(Group 3)**	VL 7 years ago	Hypopigmented spots over face. No fever, no organomegaly	+	Miltefosine for last 3 months
Sample 4 **(Group 4)**	VL 10 years ago		+	
Sample 5 **(Group 1)**	No history of Kalaazar		-	
Sample 6 **(Group 2)**	No history of fever		-	
Sample 7 **(Group 4)**	Treatment completed on July 2018 from Murarai hospital. Before treatment splenomegaly was found as 3 finger, Hb was 5.8g/dL, WBC count was 4,700.	No organomegaly	+	Treatment with Miltefosine completed on July 2018
Sample 8 **(Group 1)**		Apparently healthy		
Sample 9 **(Group 1)**		Apparently healthy		

Group 1 – Healthy Individuals
Group 2 – rK39- with lesions
Group 3 – rK39+ with lesions and under Miltefosine treatment
Group 4 – rK39+ without lesion, no treatment

Supplementary Table 1: Characteristics of blood samples from human subjects
